# PRESERFLO™ MicroShunt as a treatment option for highly increased intraocular pressure in primary open angle glaucoma and pseudoexfoliation glaucoma

**DOI:** 10.1038/s41433-025-03843-w

**Published:** 2025-05-19

**Authors:** Theresa Theilig, Menelaos Papadimitriou, Daniel Meller, Somar M. Hasan

**Affiliations:** 1 Department of Ophthalmology, University Hospital Jena, Am Klinikum 1, 07747 Jena, Germany; 2 Department of Ophthalmology, University Hospital Mannheim Medical Faculty Mannheim, University of Heidelberg Theodor-Kutzer-Ufer 1-3, 68167 Mannheim, Germany

**Keywords:** Glaucoma, Outcomes research

## Abstract

**Purpose:**

The Preserflo-MicroShunt (PF) is an established device for treating glaucoma, effectively reducing intraocular pressure (IOP) with a good safety record. However, its efficacy in eyes with significantly elevated preoperative IOP levels is not well-documented due to exclusion from many clinical studies. This study aims to evaluate PF outcomes in eyes with highly increased IOP and compare them with those having moderately increased levels.

**Methods:**

Retrospectively, eyes diagnosed with primary open angle glaucoma (POAG) or pseudoexfoliation glaucoma (PXG) undergoing PF were analysed. They were categorized into two groups: highly increased IOP (HI-IOP, ≥30 mmHg) and moderately increased IOP (MI-IOP, ≤25 mmHg). IOP, number of IOP lowering medications (NoM), success rates (SR), and postoperative complications were compared at 1, 3, 6, and 12 months.

**Results:**

One year postoperatively, IOP was reduced from 39.0 ± 7.4 mmHg to 14.4 ± 4.4 mmHg in the HI-IOP and from 19.5 ± 3.6 mmHg to 14.0 ± 4.5 mmHg in the MI-IOP group. NoM decreased significantly in both groups (*p* < 0.001 for all). Success rates did not differ between groups during the first year (*p* > 0.05 in all subgroups). However, the HI- IOP group had higher rates of persistent hypotony (7% vs. 0%, *p* = 0.02) and tendentially higher rates of choroidal detachment (23% vs. 11%, *p* = 0.08). Other adverse events were rare and comparable. Further glaucoma surgery was required in 26% and 19% of cases, respectively (*p* = 0.41).

**Conclusion:**

PF appears to be a suitable option for managing both highly and moderately increased IOP levels in POAG and PXG eyes, with comparable outcomes up to 12 months following surgery.

## Introduction

Glaucoma is the major cause of irreversible blindness worldwide [1]. The only modifiable risk factor for disease progression is the intraocular pressure (IOP). Lowering IOP starts with topical medications and laser treatment. However, many patients still need to undergo surgical IOP lowering procedures when other treatment options fail to sufficiently reduce IOP. The gold standard of glaucoma surgery is still trabeculectomy, even after many decades of its introduction by Cairns in the 1960s [2]. Multiple techniques for other filtering surgeries were introduced in the last years with variable success and complications rates. The PRESERFLO® MicroShunt (Santen Pharmaceutical Co. Ltd., Osaka, Japan) is a relatively recent Microshunt composed of styrene-block-isobutylene-block-styrene (SIBS) and can be implanted using an ab externo approach. It is 8.5 mm long with an internal lumen of 70 µm and an outer diameter of 350 µm. The lumen diameter was approximated to provide sufficient resistance to limit hypotony given published aqueous humour flow rates and viscosity [3]. However, recent data based on scanning electron microscope analysis and flow measurements using a gravity-flow setup report a much lower pressure differential, not enough to avoid hypotony [4]. Studies have demonstrated its high efficacy in reducing IOP while maintaining a favourable safety profile [5–10].

According to the Hagen-Poiseuille equation, increased preoperative IOP should result in increased flow rate through the stent, as the resistance of the stent is not affected by the pressure gradient. It is however not clear, if the increased flow rate affects the maturation of the filtering bleb and the absorption capabilities of the conjunctiva in the bleb area [11, 12]. Eyes with highly increased IOP were often excluded in prospective studies [5, 6]. In retrospective studies, most available data report results of eyes with moderately increased IOP and mean IOP levels under 25 mmHg [9, 10]. The aim of this study was to examine the results of PRESERFLO in eyes with primary open angle glaucoma (POAG) and pseudoexfoliation glaucoma (PXG) with highly increased IOP levels and to compare them with those with moderately increased IOP levels. This information is important for the patient counselling and for surgical planning in cases of highly increased IOP.

## Materials and methods

In this retrospective study, we consecutively included eyes that had undergone the implantation of PRESERFLO for uncontrolled glaucoma at the Department of Ophthalmology, Jena University Hospital in Germany. Surgeries were performed by two surgeons: Somar M. Hasan and Daniel Meller using the same described standard technique. The inclusion criteria comprised patients diagnosed with POAG or PXG and received PRESERFLO with Mitomycin C (MMC). The indication for surgery was made when a progression of visual field parameters was observed or in case of high risk of progression along with IOP levels above the target pressure (which was previously set by glaucoma specialist) under maximally tolerated local glaucoma medication. Eyes with other types of glaucoma were excluded. Included eyes were classified according to their preoperative medicated IOP in one of two groups: group 1: eyes with an IOP of ≥30 mmHg (highly increased IOP group, HI-IOP) and group 2 with those eyes of a preoperative IOP of ≤25 mmHg (moderately increased IOP group, MI-IOP). Eyes with and IOP between 26 and 29 mmHg were excluded to give a better separation between both groups.

Preoperative demographic data included age, gender, laterality, lens status, and ocular surgical history. IOP and Number of glaucoma medications (NoM) were also documented at the time of surgical indication. Postoperatively, IOP and NoM were documented at discharge (2–3 days after surgery) and on 1, 3, 6, and 12 months after surgery along with intra- and post-operative complications and re-operations at each of these time points.

Surgical success rates were categorized based on postoperative IOP levels (≤21, ≤18, ≤15 mmHg) along with an IOP reduction of at least 20% to the preoperative IOP. Complete success (CS) was defined as achieving these criteria without medication, while qualified success (QS) involved medication not exceeding the number of medication used preoperatively. Failure was also considered in case of an additional glaucoma surgery, persistent hypotony (≤5 mmHg), or loss of light perception. The need for bleb revision or needling was also considered as a failure. Success rates were calculated on month 3, 6, and 12 and compared between both groups.

The study adhered to the Declaration of Helsinki guidelines. Statistical analysis, conducted with SPSS Statistics 27.0 (IBM Corporation, Armonk, NY, USA), employed chi-square tests for success rates and patients’ characteristics, Log Rank test for survival analysis, and dependent and independent t-tests for IOP and NoM within and between the two groups, with significance set at *p* < 0.05.

### Surgical technique

Four weeks before surgery, local IOP lowering medications were stopped, and 1 week before surgery dexamethasone eyedrops (Dexapos COMOD 1.0 mg/ml eye drops, Ursapharm, Saarbrücken, Germany) were administered three times daily. Preoperative IOP levels were managed using oral Acetazolamide 250 mg (Glaupax® 250 mg tablets, Omni-Vision, Puchheim, Germany).

During the surgery, the surgical field was disinfected and exposed using a corneal fixation suture (7-0 Vicryl, Ethicon, Somerville, NJ, USA). Subsequently, the conjunctiva was opened by performing a peritomy over 2 clock hours with 2 radial cuts. Tenon was dissected from the sclera, and episcleral vessels were cautiously cauterized. MMC (0.2 mg/ml, as surgeons’ preference at the time these surgeries performed) was applied under the tenon tissue using two Lasik corneal shields for 2–3 min and then rinsed out with at least 20 ml balanced salt solution. The sclera was marked 3 mm behind the limbus, and a 2 mm long scleral tunnel was created with the provided knife. A 25-Gauge needle was then inserted into the anterior chamber through the tunnel aiming to keep an appropriate distance to the corneal endothelium. The PRESERFLO-MicroShunt was inserted through the tunnel into the anterior chamber, and drainage was checked. The tenon tissue was repositioned anteriorly and attached to the sclera with two interrupted sutures (10-0 Vicryl). The conjunctiva was closed in a water tight manner with two to four interrupted sutures (10-0 Vicryl).

After the surgery, patients received ofloxacin eye drops five times a day for one month (Floxal® 3 mg/ml eye drops, Bausch&Lomb, Laval, Canada) and dexamethasone eye drops (Dexapos COMOD, URSAPHARM, Saarbrücken, Germany) every 2 hours for one week, followed by five times a day with a monthly reduction of one drop.

## Results

123 eyes from 87 patients were included, 43 in the HI-IOP group and 80 in the MI-IOP group. Demographic data are shown in Table 1. Preoperative age, sex, laterality, lens status and NoM were comparable between both groups. The HI-IOP group included significantly more eyes with PXG (40 vs 5%) than the MI-IOP group. Significant differences can be found in preoperative IOP (Mean ± SD: 39.0 ± 7.4 versus 19.48 ± 3.6 mmHg in the HI-IOP versus MI-IOP group) and surgical history with a higher proportion of eyes undergone prior non glaucoma ocular surgery in the HI-IOP group (*p* = 0.047).

**Table 1:** Demographic data and patients’ characteristics, specification as mean ± standard deviation or number in absolute and relative terms.

		HI-IOP group (*n* = 43)		MI-IOP group (*n* = 80)		*p*-value
Age (years)		73.1	±10.4	70.4	±9.9	0.161^a^
Sex	Males	16	(37%)	27	(34%)	0.701^b^
	Females	27	(63%)	53	(66%)	
Laterality	Right	23	(53%)	41	(51%)	0.813^b^
	Left	20	(47%)	39	(49%)	
Glaucoma diagnosis	POWG	26	(60%)	76	(95%)	<0.001^b^
	PXG	17	(40%)	4	(5%)	
Prior surgery	None	16	(37%)	37	(46%)	0.047^b^
	Pseudophakia	22	(51%)	42	(52%)	
	OPs without Conjunctival participation	1	(2%)	0	(0%)	
	OPs With conjunctival participation	10	(23%)	7	(9%)	
Prior glaucoma surgery	None	31	(72%)	69	(86%)	0.243^b^
	Trabeculectomy	4	(9%)	3	(4%)	
	XEN SLT/LTP	3 3	(7%) (7%)	7 1	(9%) (1%)	
MMC application time (minutes)		2.5	±0.6	2.3	±0.6	0.087^a^
IOP (mmHg)		39.0	±7.4	19.5	±3.6	<0.001^a^
NoM		2.8	±1.4	2.9	±1.3	0.816^a^

*HI-IOP* highly increased intraocular pressure, *MI-IOP* moderately increased intraocular pressure, *POWG* primary open angle glaucoma, *PXG* pseudoexfoliation glaucoma, *XEN* XEN-Gel-Stent, *SLT* selective laser trabeculoplasty, *LTP* laser trabeculoplasty, *MMC* Mitomycin-C, *NoM* number of medication.

^a^Independent t-test.

^b^Chi-square test.

**Fig. 1:**
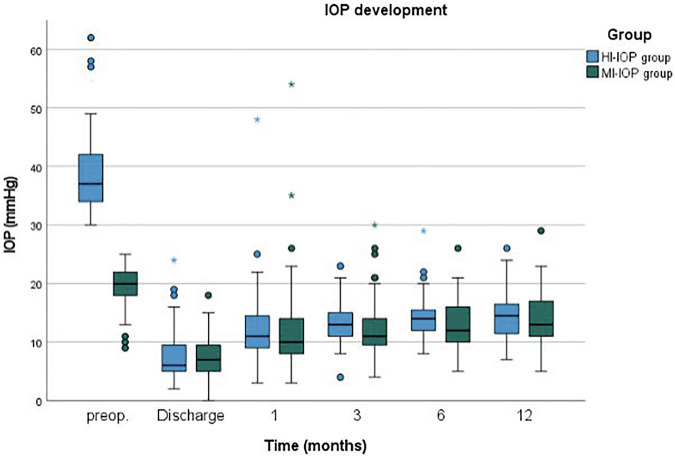
IOP development from the indication of PRESERFLO and postoperatively at discharge, and after 1, 3, and 6 months. IOP intraocular pressure, HI-IOP highly increased intraocular pressure, MI-IOP moderately increased intraocular pressure.

Eyes that underwent a second glaucoma surgery, bleb revision, or needling were classified as failures and excluded from further IOP and NoM measurements. The results of postoperative IOP and NoM can be found in Table 2. In both groups a significant IOP reduction was achieved at all time points (at discharge, at 1, 3, 6, and 12 months, *p* < 0.001 for all, Fig. 1). Postoperative IOP levels did not differ between both groups at any time point (*p* > 0.05 for all). The relative and absolute IOP reduction was significantly higher in the HI-IOP group compared to the MI-IOP group (at 12 months: 62.1% vs. 27.5%, *p* < 0.001; 24.9 mmHg vs. 5.9 mmHg, *p* < 0.001; respectively).

**Table 2:** IOP and NoM preoperatively, and postoperatively at discharge, and after 1, 3, 6 and 12 months; SD (standard deviation).

		HI-IOP group			MI-IOP group			
	Visit	Mean	±SD	*p*-value^a^	Mean	±SD	*p*-value^a^	*p*-value^b^
IOP (mmHg)	preop.	39.0	±7.4		19.48	±3.6		<0.001
	Discharge	7.8	±4.7	<0.001	7.3	±3.3	<0.001	0.501
	1 month	12.8	±7.7	<0.001	12.0	±7.8	<0.001	0.617
	3 months	13.8	±4.3	<0.001	12.4	±5.1	<0.001	0.157
	6 months	14.4	±4.6	<0.001	13.2	±4.4	<0.001	0.251
	12 months	14.4	±4.4	<0.001	14.0	±4.4	<0.001	0.664
NoM	preop.	2.8	±1.4		2.9	±1.3		0.816
	Discharge	0.0	±0.2	<0.001	0.0	±0.0	<0.001	0.323
	1 month	0.0	±0.2	<0.001	0.1	±0.5	<0.001	0.521
	3 months	0.1	±0.6	<0.001	0.2	±0.6	<0.001	0.829
	6 months	0.1	±0.5	<0.001	0.1	±0.5	<0.001	0.954
	12 months	0.2	±0.6	<0.001	0.5	±1.1	<0.001	0.085

*HI-IOP* highly increased intraocular pressure, *MI-IOP* moderately increased intraocular pressure, *NoM* number of medications.

^a^Dependent t-test related to preoperative values.

^b^Independent t-test between HI-IOP group and MI-IOP group.

The NoM was reduced significantly in both groups at all time points (*p* < 0.001 for all). Intergroup comparison did not show any difference at any time point (*p* > 0.05) but with a tendency towards higher NoM at 12 months in the MI-IOP group (*p* = 0.085).

### Success rates

**Fig. 2:**
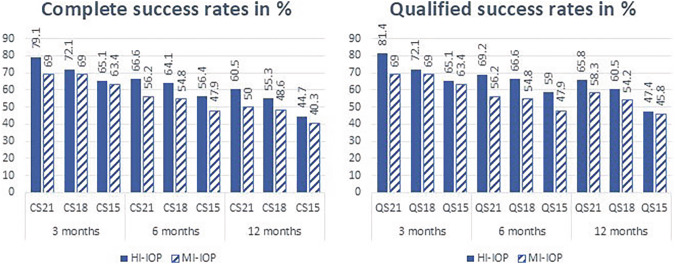
Success rates at 3-, 6-, and 12-months comparing HI-IOP-group and MI-IOP-group, based on postoperative IOP levels (≤21, ≤18, ≤15 mmHg) (*p* > 0.05 for all). CS complete success, IOP intraocular pressure, HI-IOP highly increased intraocular pressure, MI-IOP moderately increased intraocular pressure, QS qualified success, the number indicates the IOP limit of success.

The rates of Complete Success in the HI-IOP group after 12 months were by 60.5% in the CS21, 55.3% in the CS18 and by 44.7% in the CS15 group. Those of Qualified Success were by 65.8% in the QS21, 60.5% in the QS18 and by 47.4% in the QS15 groups. In the MI-IOP group the Complete Success rates were by 50.0%, 48.6% and 40.3% in the CS21, CS18 and CS15 groups, respectively. Rates of Qualified Success in 12 months were by 59.7%, 55.5% and 47.2% in the QS21, QS18 and QS15 groups, respectively. A list of all calculated success rates after 3, 6, and the 12 months can be found in Fig. 2. The survival curves are shown in Supplementary Fig. 1. There was no significant difference of complete or qualified success rates between the HI-IOP and the MI-IOP groups at any time point (*p* > 0.05 for all).

In subgroup analyses PXG and POAG were compared within the HI-IOP and MI-IOP ranges, as well as the HI-IOP and MI-IOP groups each compared only with the eyes of the individual diagnoses (PXG or POAG). No significant differences were found in any of the groups regarding the success rates, IOP- and NoM-development (*p* > 0.05 for all).

An additional glaucoma surgery was performed in 10 eyes (23%) in the HI-IOP group within the first 12 months compared to 14 eyes (18%) in the MI-IOP (*p* = 0.41). In all these cases an open bleb revision was performed.

The adverse events were mild and could be treated mainly conservatively (Table 3). 3 cases of persistent postoperative hypotony (defined as IOP < 6 mmHg over ≥30 days) occurred in the HI-IOP group compared to 0 in the MI-IOP group (*p* = 0.017). All these cases resolved without surgical intervention (after 55, 145, and 219 days). Choroidal detachments were seen twice as often in the HI-IOP group compared to the MI-IOP group (10 eyes (23.3%) vs. 9 eyes (11.3%), *p* = 0.08); a tendency towards statistical significance was observed. In the above mentioned cases of persistent hypotony choroidal detachments resolved after 55, 47 and 114 days, without any further intervention. We observed no case of hypotonic maculopathy in the whole cohort. Other complications did not differ between both groups.

**Table 3:** Postoperative complications and re-operations, chi-square test.

	HI-IOP group (*n* = 43)		MI-IOP group (*n* = 80)		*p*-value
Postoperative hypotony (≤5 mmHg)	21	(49%)	29	(40%)	0.345
Persistent hypotony	3	(7%)	0	(0%)	0.017
Choroidal detachment	10	(23%)	9	(11%)	0.079
Hyphaema	6	(14%)	11	(14%)	0.975
Aqueous misdirection syndrome	0	(0%)	2	(2%)	0.296
Haemorrhagic choroidal detachment	1	(2%)	0	(0%)	0.171
Positive Seidel test	0	(0%)	5	(6%)	0.094
Fibrin reaction in anterior chamber	2	(5%)	0	(0%)	0.052
Irvine-Gass-Syndrome	1	(2%)	1	(1%)	0.653
Non-IOP-lowering surgeries	2	(2%)	8	(10%)	0.302
IOP-lowering surgery	10	(23%)	14	(18%)	0.407

*HI-IOP* highly increased intraocular pressure, *MI-IOP* moderately increased intraocular pressure.

Other non-IOP-lowering surgeries were performed as following: in the HI-IOP group 1 cataract surgery and 1 triple penetrating keratoplasty (in a case of already preoperative documented corneal decompensation); in the MI-IOP group 4 cataract surgeries along with 2 eyes which received intravitreal injections because of diabetic macular oedema or persistent Irvine-Gass-Syndrome, 2 eyes received iridectomy and core vitrectomy because of aqueous misdirection syndrome. There was no significant difference between both groups regarding the number of non-IOP-lowering re-operations.

## Discussion

PRESERFLO MicroShunt is a novel surgical glaucoma device which provides an effective reduction of IOP and NoM. As part of the MIBS, it is less invasive than trabeculectomy as no preparation of scleral flap, no scleral sutures and no iridectomy is needed. As a result, Preserflo needs less postoperative interventions and leads to less adverse events [6, 8]. Success rates were slightly lower than those of trabeculectomy, especially in the lower IOP levels [13]. Still, some authors found comparable efficacy to trabeculectomy, even on the long term [6–8, 14].

As the resistance of the stent is defined through its length and lumen diameter, increased preoperative IOP should result in increased flow rates through the distal orifice. This increased flow should be accompanied by an increased absorption capacity of the conjunctival bleb. However, in many prospective studies eyes with highly increased IOP levels were excluded [5, 6]. Most retrospective studies report results of eyes with moderately increased IOP levels, so that the current available data offers only limited evidence about justified indication of PRESERFLO in eyes with highly increased IOP levels. To the best of our knowledge, this is the first study examining particularly this group of eyes and comparing those to a group with moderately increased IOP.

In our study a significant IOP reduction was achieved in both examined groups (*p* < 0.001) and postoperative IOP levels did not differ between both groups at any time point within the first year. However, the relative and absolute IOP reduction was (as expected) higher in the HI-IOP compared to the MI-IOP group (62.1% vs. 27.5%; 24.9 mmHg vs. 5.9 mmHg; respectively). Our postoperative results are comparable to available data where postoperative IOP levels ranged from 10.7 to 14.6 mmHg, (absolute reduction: 6.8–13.1 mmHg, relative reduction: 32–55%) 1 year post surgery [5, 7, 9, 10, 15–17]. As current literature reports a mixture of moderate and high IOP levels, our above mentioned absolute and relative IOP reduction frame the already reported data [15, 16]. According to this, the achieved postoperative IOP levels seem to be independent of the preoperative IOP levels. The current data shows varying results regarding preoperative IOD in relation to postoperative success. In some studies higher baseline IOP has been shown to be a predictive factor of surgical success following PRESERFLO [12], but also following larger drainage implants [18, 19]. One reason might be the failure to achieve the 20% reduction of IOP in cases of only moderately increased baseline IOP, as IOPs in the low teens are less possible on the long-term following PRESERFLO or drainage implants. Our study, however, shows comparable results in groups of highly and moderately increased IOP. This discrepancy might be the result of different study designs: while we performed a direct comparison between eyes with baseline IOP ≥ 30 or ≤25 mmHg, mentioned study based on regression analysis to correlate baseline IOP with success rates. Additionally, differences in inclusion criteria may contribute, as patients with IOPs >40 or 45 mmHg were excluded from that study, while our study allowed these patients to be included. On the other hand in the mega-analysis of de Francesco et al. higher baseline IOP was associated with an increased risk of failure [20]. The comparability of the mentioned study to the one by Ibarz et al. is limited due to the stricter success criteria (IOP 6–14 mmHg postoperatively) applied while studying risk factors for surgical failure. Moreover, in this mega-analysis several studies with different study designs, different postoperative therapy regimes in the individual study centres, different MMC concentrations and application times could have an influence on the comparability of the results.

The special design of the implant, based on its length and the size of lumen is supposed to prevent postoperative hypotony [3], although, in-vitro data estimate a pressure differential across the device to be as low as 2.6 mmHg, which is below numerical hypotony. However mid-term and long-term postoperative IOP levels are mainly determined by the development of the bleb and the ongoing scarring process in the filtration zone [21]. The structural changes of the bleb occur as part of the postoperative wound healing process, which is influenced by multiple factors. Inflammation and associated pro-inflammatory cytokines, especially transforming growth factor β (TGF-β), lead to transdifferentiation of fibroblasts to myofibroblasts, that are highly contractile and express extracellular matrix proteins [22]. As Freedman reported, concentration of TGF-β in the aqueous humour is higher in eyes with elevated IOP, which could pose a special risk on those eyes with preoperative highly increased IOP [23]. Increased flow rate of aqueous correlated with increased preoperative IOP might accordingly result in higher exposure of the filtration bleb to inflammatory mediators, especially the TGF-β. The increased flow rate associated with higher preoperative IOP might also result in increased pressure inside the forming bleb, if this exceeds the capillary driving pressure, it might negatively affect the maturation of the bleb [24]. Moreover, increased flow-rate might be an inducing factor for the shear stress inside the bleb, interstitial fluid flow can modulate fibrosis by increased TGF-β expression caused by shear stress via mechanotransduction [25, 26]. According to our results which showed comparable outcomes of the different IOP values, the above-mentioned possible risks seem to play a secondary role. Anti-inflammatory and antiproliferative prevention by peri- and postoperative topical steroid therapy and the use of antimetabolites appear to play an important role in contradicting the effect of these factors. Accordingly significantly lower IOP levels were achieved by using higher concentration of MMC following PRESERFLO (0.4 vs. 0.2 mg/ml) [5, 8, 20]. In this context it should also be noted, that the preoperatively prescribed Acetazolamid suppresses the aqueous production, possibly leading to lower IOP levels directly prior to the surgery, which could possibly weaken the above discussed effects in the very early postoperative phase. Nevertheless, eyes with preoperatively highly increased IOP would still potentially have a tendency towards higher IOPs after the effect of the drug wears off, leading to the already described higher fluid flow-rate through the implant and also the bleb.

Regarding the success rates, both groups did not show significant differences at any time point in all IOP gradings. Our success rates are comparable with the results of Baker (CS17 53.9%, QS17 66.1%) [6], Martinez (CS 62.1%, QS 82.8% after 12 months as solo procedure) [16] and Scheres (CS18 58%, QS18 79%) [27]. In other studies, the authors report higher success rates at 12 months (Batlle QS14 100% [15], Jamke QS18 87% [28], respectively). The differences in the success rates are probably mainly caused by the different selection criteria and the operative management. Batlle and Jamke both excluded eyes with previous incisional surgery whereas in our cohort previous surgery was not an exclusion criterion. Moreover, the MMC dosage in the different studies is not always the same. We used 0.2 mg/ml; higher success rates were reported with higher MMC dosage [5, 8]. Most of our failures are due to IOP-lowering re-surgeries (76.9% in the HI-IOP group and 70% in the MI-IOP group) as a result of primary bleb failure. Intra- and postoperative complications were comparable between both groups. These were mild and mainly managed conservatively.

One of the important observations of our study was the increased rate of chronic hypotony (7.0% vs. 0%, *p* = 0.017) and choroidal detachments (23% vs 11%, *p* = 0.08) in the HI-IOP compared to the MI-IOP group, although the rate of postoperative hypotony was comparable between both groups (*p* = 0.268). This increased rate of choroidal detachments despite comparable hypotony rates is most likely a result of the abrupt reduction of IOP levels in this group. In eyes that have undergone trabeculectomy, higher IOP-reduction or rather higher preoperative IOP levels are already described as risk factors for choroidal detachment [29, 30] beside exfoliation glaucoma, lower postoperative IOP, older age and thicker cornea [29, 31, 32]. Although the rates of hypotonic maculopathy were comparable between both groups, it might be appropriate to consider surgical modifications in these cases of HI-IOP to contradict the expected sudden reduction of pressure such as the use of 10-0 Ethylon suture as a releasable ripcord, similar to non-valved glaucoma drainage devices, which can be removed later.

The main limitations of our study are the retrospective design and the short follow-up-time. Although baseline characteristics did not differ regarding age, gender and NoM, we had a higher rate of PXG in the HI-IOP group along with more eyes that had undergone prior surgeries than in the MI-IOP group. PXG seems however not to affect the results of PRESERFLO compared to POAG [33]. On the other hand, previous surgery is a known risk factor for failure in filtering surgery [22]. The fact that IOP levels and SUCCESS RATES did not differ between both groups despite these differences supports the efficacy of PRESERFLO in eyes with HI-IOP.

In conclusion, PRESERFLO seems to be an effective surgical management of POAG and PXG in both eyes with highly and moderately increased IOP levels and was able to reduce IOP and NoM to comparable levels along with similar safety profile. The postoperative IOP seems not to be affected by the preoperative IOP. Studies with longer follow up times are still needed to confirm these results on the long term.

## Summary

### What was known before:

- The Preserflo-MicroShunt is a widely recognized glaucoma device with a good safety profile.
- The Preserflo-MicroShunt effectively reduces intraocular pressure (IOP), but results in highly increased (≥30 mmHg) preoperative IOP levels are limited.

### What this study adds:

- This study demonstrates that postoperative outcomes, including IOP, the number of IOP-lowering medications, and success rates, are similar in eyes with highly and moderately increased preoperative IOP.
- Eyes with highly increased preoperative IOP undergoing PRESERFLO implantation might be more prone to persistent hypotony and may exhibit a higher incidence of choroidal detachments.

## Supplementary information

Supplemental Figure 1

Supplemental Figure 1 Legend

## Data Availability

The datasets generated during the current study are available from the corresponding author on reasonable request.
