# Supplementary figures and images for: PRESERFLO™ MicroShunt as a treatment option for highly increased intraocular pressure in primary open angle glaucoma and pseudoexfoliation glaucoma
Source: Eye (Lond). 2025 May 19;39(11):2253–9. doi: 10.1038/s41433-025-03843-w (PMC12274334; doi:10.1038/s41433-025-03843-w)

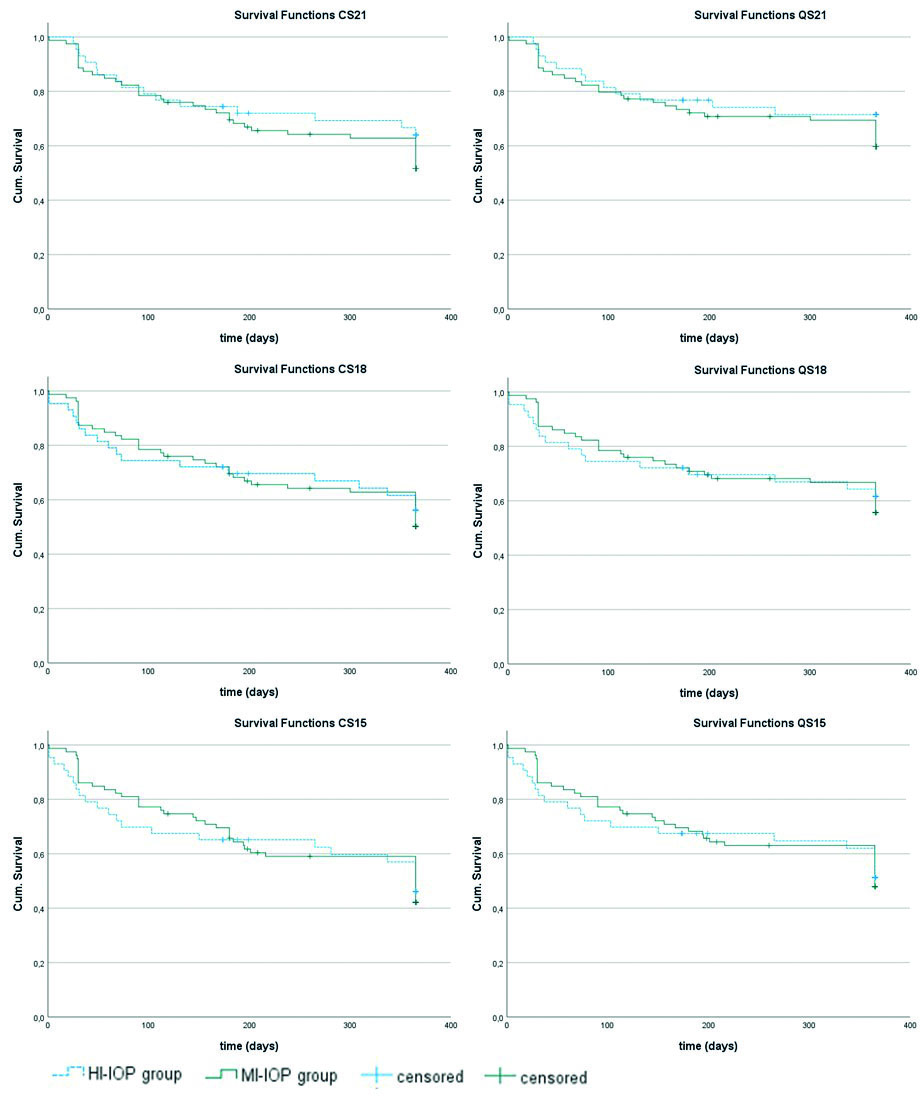

Supplement: Supplementary file 1 — Supplemental Figure 1 [file 41433_2025_3843_MOESM1_ESM.jpg]
