# Supplementary material for: PRESERFLO™ MicroShunt as a treatment option for highly increased intraocular pressure in primary open angle glaucoma and pseudoexfoliation glaucoma
Source: Eye (Lond). 2025 May 19;39(11):2253–9. doi: 10.1038/s41433-025-03843-w (PMC12274334; doi:10.1038/s41433-025-03843-w)
Supplement: Supplementary file 2 — Supplemental Figure 1 Legend [file 41433_2025_3843_MOESM2_ESM.docx]

Supplemental Figure 1

Survival curves of complete and qualified success comparing HI-IOP-group and MI-IOP-group, based on postoperative IOP levels (≤21, ≤18, ≤15mmHg) (p>0.05 for all, Log Rank test). Abbreviations: CS: complete success, IOP: intraocular pressure. HI-IOP: highly increased intraocular pressure, MI-IOP moderately increased intraocular pressure, QS: qualified success, the number indicates the IOP limit of success
